# Current Status and Advances in Quantitative Proteomic Mass Spectrometry

**DOI:** 10.1155/2013/180605

**Published:** 2013-03-06

**Authors:** Valerie C. Wasinger, Ming Zeng, Yunki Yau

**Affiliations:** ^1^Bioanalytical Mass Spectrometry Facility, Mark Wainwright Analytical Centre, The University of New South Wales, Sydney, NSW 2052, Australia; ^2^Department of Gastroenterology and Liver Services, Concord Repatriation General Hospital, Sydeny, NSW 2139, Australia

## Abstract

The accurate quantitation of proteins and peptides in complex biological systems is one of the most challenging areas of proteomics. Mass spectrometry-based approaches have forged significant in-roads allowing accurate and sensitive quantitation and the ability to multiplex vastly complex samples through the application of robust bioinformatic tools. These relative and absolute quantitative measures using label-free, tags, or stable isotope labelling have their own strengths and limitations. The continuous development of these methods is vital for increasing reproducibility in the rapidly expanding application of quantitative proteomics in biomarker discovery and validation. This paper provides a critical overview of the primary mass spectrometry-based quantitative approaches and the current status of quantitative proteomics in biomedical research.

## 1. Introduction

Quantification in a proteomics setting relies on the ability to detect small changes in protein and peptide abundance in response to an altered state [1]. Differential analysis is generated from LC-MS experiments and can be carried out using both label and label-free approaches. For trace amounts of proteins within complex proteomes such as plasma, tears, and urine, no singular technique should be used as a stand-alone guarantee of quantitative precision without hypothesis-driven, targeted approaches. Enrichment and fractionation of specific classes of protein is beneficial during the discovery phase of a project, but because these methods can involve numerous steps, they can become a limiting factor for large scale validation. The variability introduced by multiple methods prior to quantitative mass spectrometry should be assessed, and it is paramount that protein measurements reflect the authentic concentration in the original sample. The development of methods for accurate protein quantitation is one of the most challenging areas of proteomics.

Quantitative proteomics comes in two forms: absolute and relative. Relative quantitation compares the levels of a specific protein in different samples with results being expressed as a relative fold change of protein abundance [2]. Absolute quantitation is the determination of the exact amount or mass concentration of a protein, for example, in units of ng/mL of a plasma biomarker.

Traditional proteomic quantitation approaches rely on high-resolution protein separation by 2D gels. The use of dyes, fluorophores, or radioactivity to label proteins allows visualization of spots/bands with differential intensities [3, 4]. These methods facilitate relative abundance comparison but require many replicates and intensive image analysis that can often be quite user subjective. The simplicity of mass spectrometry-based approaches addresses issues of reproducibility [5] and poor representation of low-abundance [6], low-mass, and basic proteins [7, 8], as well as the need for the postdifferential identification by MS [3] as it is inherent in the separation methods. MS-based methods have also come into prominence compared to traditional antibody-based methods due to their higher specificity, good reproducibility and precision, and ability to rapidly analyse hundreds of peptide transitions in one MRM assay [9]. Pragmatically, the course of a biomarker project sees a number of quantitative techniques used from discovery-driven low-cost methods such as relative and label-free quantitation to hypothesis-driven quantitation using synthetic standards with complimentary analysis of trends by alternative techniques such as ELISA or Western blot. Here, we provide a critical overview of the main MS-based quantitation approaches and outline the advances and challenges of applying these techniques in protein biomarker discovery and validation. 

## 2. Quantitative Proteomics in Biomarker Discovery

The ultimate aim of biomarker discovery is to develop a simple differential test to be used as a clinical evaluation tool. This requires a lengthy and difficult process which involves candidate discovery, verification, validation, and translation to clinical laboratory use [12, 13]. Current discovery studies aim to detect disease-specific markers by analysing and comparing healthy controls and disease-affected subjects [14], and despite the discovery of increasing numbers of potential markers, few have progressed to clinical practice [15, 16]. Much of this dilemma is a reflection of the challenges associated with linking bench to clinic outcomes and providing basic researchers with the opportunity to finance and progress their science past the validation phase [12, 17]. The development of targeted, quantitative approaches that provide accurate and statistically reliable quantitative outcomes for multisite studies may provide a critical bridge to establishing validity of individual or panels of biomarkers.

A challenge facing biomarker development is the sheer complexity and range of concentrations within the human proteome [12, 16]. Human plasma is estimated to contain more than 10,000 core proteins [35], of which only small fractions are effectively characterized with current technology [36]. Proteins in plasma have a 10^12^-fold concentration range, from millimolar for albumin, down to attomolar ranges, and further for cytokines [35] and other proteins, hormones, and peptides. This greatly exceeds the ability of current proteomic approaches, which have linearity over ~3 orders of magnitude [16]. 

**Figure 1:**
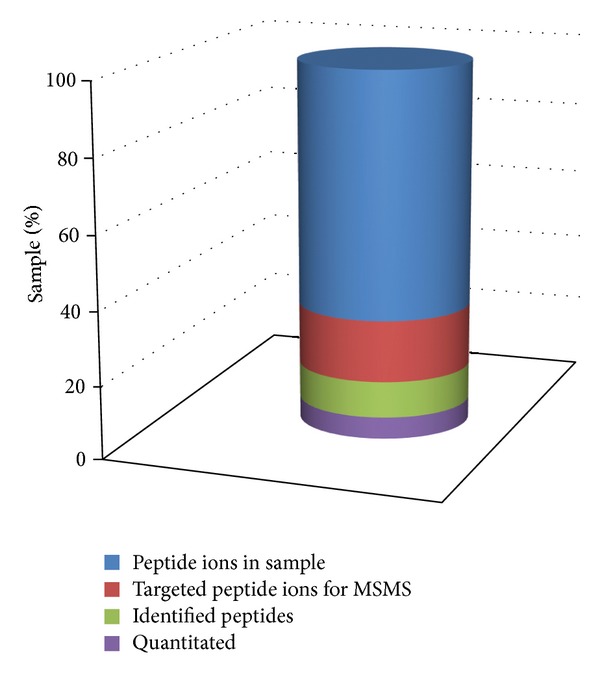
Relationship between the peptide ion content and the difficulty in obtaining sufficient MSMS information to both identify and also quantitate those peptides. Adapted from Michalski et al. [10] and Liu et al. [11].

Disease-specific proteins, including low-mass peptides, can be low in abundance and difficult to detect amongst a diverse “sea” of proteins [37]. Combined with the immense extent of human and disease variation and the challenges facing the development of sensitive and specific differentiators, developing these technologies to the clinic is a formidable task. Discovery phase quantitative approaches entail the differentiation of as many peptides as possible (rather than the identification of all proteins) from LC-MS experiments and is highly dependent on scan speed, sensitivity, and ability to isolate precursor ions for selection to MS/MS [10]. Figure 1 shows the relationship between peptide ions and quantitation and is adapted from Michalski et al. [10] and Liu et al. [11]. This figure demonstrates the gap between peptide content and ability to quantitate those peptides and proteins comprehensively to provide quantitative coverage. As instruments improve in these areas, there will be an associated increase in depth of coverage and accuracy which is required to discern the very small changes in abundance, peptide modifications, and mass differences that delineate a disease type or process. For targeted approaches, the use of high-resolution instruments has the advantage of relying on the mass accuracy to provide fewer transitions and therefore being able to simultaneously monitor more peptides within the one scheduled experiment. This should assist the reliability and precision of targeted assays to unambiguously identify the target peptide and avoid interfering transitions particularly in complex biological matrices [38]. Indeed, there is a growing consensus that panels of multiple biomarkers are more likely to achieve adequate clinical sensitivity and specificity [12, 37, 39].

There are a number of novel techniques that allow for the fractionation, depletion, enrichment, and equalisation of complex samples to assist in improving the proteome coverage and number of peptide ions targeted for MS/MS within an instrument's detection range. Fractionation techniques can be applied to cut samples into subgroups of fewer proteins [15] and are most commonly in the form of (gel) electrophoresis and liquid chromatography (LC), techniques which exploit a variety of physicochemical properties of proteins to fractionate proteomes [7]. To reduce protein concentration variability, high-abundance proteins such as albumin can be removed from plasma samples through immunodepletion. There is, however, a risk of codepletion of potentially significant biomarkers due to nonspecific binding or loss of biomarkers bound to higher-abundance carrier proteins [40–42]. These techniques in combination effectively allow the detection of trace proteins [7, 15, 16]. However, any additional manipulation during the sample processing can introduce preanalytical variables that cause changes in quantitative peptide amounts [9]. While the previous techniques can improve discovery of trace levels of candidate protein biomarkers, extensive validation and standardization of these steps will be required before they can be used for direct clinical applications [9, 43].

Data analysis is yet another significant challenge associated with MS-based proteomics. With the enormous volumes of proteomic data generated, expert manual analysis would be inconsistent and unfeasible [44]. Thus, bioinformatics tools are crucial in the determination of which proteins and peptides emerge as candidate biomarkers from discovery studies and the interpretation of quantitative data [9, 45]. There is a need for sophisticated yet transparent computational methods and algorithms to allow for consistent analysis and interpretation of proteomic data using statistical principles [45]. The development and validation of such tools is a critical part in the process of developing quality standards for MS experiments and, hence, generating reproducible and accurate data.

## 3. Strengths and Limitations of Mass Spectrometry-Based Quantitative Approaches

Protein mass spectrometry is not inherently quantitative. There are many reasons as to why the amount of analyte compared to the MS signal intensity does not always show a linear relationship [3, 44]. Because of this, accurate comparisons between two samples must be based on the same individual peptide in LC-MS/MS experiments conducted under the same conditions [4], particularly for absolute quantitation. Table 1 presents an overview of the technical parameters of the main quantitative approaches, their strengths and limitations.

### 3.1. Label-Free Approaches

Two widely used label-free quantitative methods are spectral counting and peptide peak intensity measurement. Spectral counting requires proteins to have sufficient peptides (both in number and abundance) to trigger MS/MS data for quantification and identification. The approach is based on the observation that more abundant proteins will produce more MS/MS spectra than less abundant proteins, and abundant peptides are sampled more often in fragment ion scans than are low abundance peptides. Relative quantitation by spectral count thus involves comparing the number of identified spectra from the same protein between different samples [54]. Spectral counting is a protein-centric approach that is less reliable for trace and/or low mass proteins; and less responsive toward small changes in response (<2 orders of magnitude) [11, 55], favoring higher abundance “average” proteins [2], while lower identification rates for proteins with low sequence coverage and nontryptic or fewer peptides are a consequence of the methods used for identification as much as the dynamic range of the sample and the limited duty cycle of the MS instrument [56]. This approach has been modified into forms such as the exponentially modified protein abundance index [57] and absolute protein expression profiling [58]. 

Relative quantitation using peptide peak intensity measurements involves comparing the MS peptide ion intensities belonging to a given protein [59]. The ion chromatograms for every peptide are extracted from an LC-MS run, and their peak areas are integrated over the chromatographic time scale. These values can be compared to respective values in other experiments for relative quantitation, and only the same ion species can be compared between different samples. Hence, this approach requires multiple replicates and correlation of retention time with m/z ion features and charge state to avoid discrepancy in matching common ions detected in each run. The coverage of common ions between different samples is strongly dependent on sample preparation and can be severely affected by column conditions, instrument sensitivity, and calibration. These variables are pronounced when running long-term projects where analysis is carried out over weeks to months and can introduce approximately 40% discrepancy at the peptide level [4]. Label-free techniques have been performed in many studies and are promising alternatives to stable isotope labeling. They are fast, easy to perform, and inexpensive, and they allow higher dynamic range [3]. Furthermore, any soluble biological material can be used, and unlimited numbers of samples can be compared [4].

### 3.2. Stable Isotope Labelling

Stable isotope labelling techniques are based on the introduction of a differential mass tag which affects only the mass of a protein or peptide without changing the chemical properties during chromatography or MS [2]. Relative or absolute quantitation can be achieved by using MS to compare the abundance of a labeled “heavy” (known concentration) against the endogenous “light” isoforms [60]. Stable isotope labels are introduced metabolically or chemically at either the protein or peptide level during sample preparation.

Metabolic labelling involves the introduction of stable isotopes to whole cells through the growth medium, which enables the labels to be incorporated during normal cell growth and division [61]. Differently labelled samples can be pooled together for subsequent preparation which avoids variability of sample preparation. However, this method is not applicable to samples that are not metabolically active such as plasma [2]. While the original ^15^N labelling can only compare two samples in one experiment, high-throughput quantitation was developed in the form of stable isotope labelling by amino acids (SILAC) [62]. SILAC incorporates heavy and light forms of arginine or lysine *in vivo* and also combines light and heavy samples prior to sample preparation to significantly reduce sample handling and thus quantitative errors, allowing very small changes in protein levels as well as protein modifications to be detected.

In chemical labelling, the isotope label is introduced to proteins or peptides by a chemical reaction, such as with isotope-coded affinity tags (ICAT) [63] and isotope-coded protein labels (ICPL) [64]. ICAT labels specifically bind to cysteine, a relatively rare amino acid, which effectively reduces sample complexity but also limits its use since it cannot track proteins that lack cysteine residues [2]. Another limitation of ICAT is that only two samples can be compared in a single analysis. 

The development of isobaric mass tags such as tandem mass tag (TMT) [77] and isobaric tags for relative and absolute quantification (iTRAQ) [78] allows for the comparison of up to eight samples in parallel [79, 80]. iTRAQ involves the introduction of mass-balanced labels at the level of tryptic peptides which produce labelled peptides of the same total mass that coelute in liquid chromatography. The different mass tags are differentiated by the mass spectrometer only upon peptide fragmentation [81]. Despite having disadvantages such as variability in labelling efficiencies and protein digestion [2], TMT and iTRAQ are favourable for quantitative biomarker discovery due to their ability to multiplex up to eight samples [82]. A summary of some recent projects is demonstrated in Table 2 and shows the variety of techniques applied for quantitation. 

### 3.3. Multiple Reaction Monitoring

Multiple reaction monitoring (MRM) is the main current approach for highly confident protein and peptide quantification. MRM targets specific peptides in complex samples by typically using a triple quadrupole mass spectrometer or hybrid triple quadrupole/linear ion trap mass spectrometer. These instruments have two mass filters that can select a predefined peptide ion and a combination of its specific fragment ions to analyse and monitor over time for accurate quantitation [2, 83]. Combinations of peptide mass and product ion masses create a unique signature for a particular peptide with increase in confidence, the more parent and product masses that are detected.

Absolute quantitation can be achieved when MRM is incorporated with isotopically labelled synthetic peptide internal standards, which are designed to be identical to target peptides [84]. For MRM using synthetic internal standards, known concentrations of heavy synthetic peptides are spiked into the sample, and the concentration of the target native peptide can be calculated by measuring the observed MRM response against a standard curve normalised by the internal heavy spike [3, 83]. 

MRM has a greater sensitivity towards low abundance peptides and relatively good quantitative precision compared to other methods discussed [85]. It is capable of detecting attomole concentrations of peptides across a dynamic range of up to 10^5^ [66, 86]. The main challenge of MRM absolute quantitation is the need for suitable internal standards to be synthesized for each target peptide. Furthermore, absolute MRM quantitation only measures the abundance of individual peptides and makes assumptions on the concentration of the whole protein. Therefore, biomarkers detected and quantified using MRM must be validated using multiple peptides from the same protein (challenging for biofluids) and additional technology to confirm the existence of the actual protein [2]. MRM remains peptide-centric for many biomarker studies.

MRM has been used to quantify major plasma proteins and target biomarkers for a range of diseases.  Table 3 lists recent studies conducted using plasma and serum for MRM-based approaches with some quantitation achieving attomolar levels of detection of peptides in one of the most complex human samples available. The MRM approach can also be used for relative quantitation without the use of stable isotopes [87]. A recent multi-site study has confirmed the reproducibility and sensitivity of MRM-based quantitation of plasma proteins [88]. MRM therefore holds great potential to be applied as a specific platform for validation of candidate biomarkers in systematic quantitative studies of clinically relevant peptides.

Further instrument developments have taken advantage of the high resolution and mass accuracy of the TOF and orbitrap analysers and combined them with the selectivity of the quad analysers by replacing the third quad with either an orbitrap or a TOF analyser. These high resolution/accurate mass (HR/AM) instruments are addressing the challenge of eliminating cofiltering interfering ions, while taking advantage of the accuracy afforded by these instruments. In experiments similar to MRM called parallel reaction monitoring (PRM), it is possible to detect all product ions of a peptide in parallel rather than just few transitions per peptide. This allows an increased number of peptides to be quantitated in the one experiment. This combination of analysers firstly uses the quadrupole to select a restricted m/z range (with broad mass filtering window typically 2–100Th, rather than broad scan of around 700Th), and the MS/MS mode provides further selectivity and accuracy utilizing the orbitrap or TOF analyser to achieve higher resolution and mass accuracy in both MS and MS/MS scanning modes [89]. A reduced mass filter window as low as 0.2Th allows reliable discrimination of targeted ions and increased sensitivity <1 ppm and mass accuracy [90]. These instruments are advancing the reliability and accuracy of quantitative proteomics and are just the beginning to a new era in quantitation that will provide inherent quantitative sampling of all peptides and their product ions in highly complex samples. 

## 4. Postdiscovery Validation Phase Platforms 

The use of multiparametric assays is becoming an increasing necessity in quantitative studies to overcome a variety of challenges associated with properties of the marker and/or the techniques including immobilisation efficiencies, detection, signal-to-noise [91]. Proteomic-based quantitation of potential biomarkers requires further validation using orthogonal techniques. This is required for both verification as much as for the routine measurement in clinical investigations [12, 13]. The gold standard for validation experiments is by enzyme-linked immunosorbent assays (ELISA). However, alternative techniques such as Western blot, fluorescent bead, chip immunoassay arrays, or Surface Plasmon Resonance (SPR) are also commonly used [91, 92]. Validation by any of these techniques is to complement the onerous requirements for clinical assays: high-throughput, high measurement precision (coefficients of variation of less than 10%) and sufficient sensitivity [92]. The recent developments in multiplexed protein immunoassays such as lateral flow immunoassays and miniaturized microassays [93] hold great promise in advancing panels of biomarkers developed from MS-based proteomics research towards clinical applications. In addition to these orthogonal approaches, parallel validation techniques involving Stable Isotope Standards and Capture by Antipeptide Antibodies (SISCAPA) [94] may also be beneficial.

## 5. Conclusion

Quantitative proteomic analysis has been a point of discussion for the last four decades, with comparative and once limited MS-based techniques heralding the advances that would forge the necessary connection between the dynamic biology of a system and its quantitative proteomic content. The major advances in quantitative MS proteomics have been exceptionally demonstrated over the last decade with the introduction of compatible and reliable label and label-free techniques. These advances now require further developments in bioinformatics and downstream validation, technologies that are required to make sense of complex data and enable researchers to infer more meaningful data that will transform into clinical benefit for years to come.

## Figures and Tables

**Table 1:** Overview of the main approaches for quantitative proteomics. Modified from Schulze and Usadel [4] and Ly and Wasinger [4, 7].

Method	Dynamic range^a^	Coverage	Quant accuracy, (throughput) R = relative, A = absolute	Associated software	Link
Label-free					
2D gels	1 to 4, stain dependent	Medium	Medium (low) R Requires MS identification.	PDQuest Progenesis SameSpots Melanie [18] Phoretix	http://www.bio-rad.com/ http://www.nonlinear.com/ http://www.genebio.com/ http://www.perkinelmer.com/
Ion intensities MS^1^	3	Good	Medium, (medium to high) R, LC dependent.	Progenesis LCMS msInspect [19] MSight [20] TOPP [21] PEPPeR [22] SuperHirn [23] DeCyder MS SIEVE ProteinLynx	http://www.nonlinear.com/ http://proteomics.fhcrc.org http://web.expasy.org http://open-ms.sourceforge.net/ http://www.broadinstitute.org/ http://www.waters.com/ http://www.gelifesciences.com/ http://thermo.com/ http://www.waters.com/
Spectrum count MS^2^	3, Inaccurate for low abundance.	Good	Poor, (medium to high) R LC dependent	Scaffold [24] Elucidator ProteoIQ	http://www.proteomesoftware.com/ http://www.rosettabio.com/ http://www.bioinquire.com/
APEX,emPAI	3 or 4	Good	Poor, (high) R,within sample only.	APEX [25] Mascot	http://pfgrc.jcvi.org/index.php/bioinformatics/apex.html http://www.matrixscience.com/
Metabolic labeling					
^15^N	1 to 2	Medium	Precise, (low). R, between 2 conditions.	Scaffold MSQuant [26]	http://www.proteomesoftware.com/ http://msquant.sourceforge.net/
SILAC	1 to 2	Medium	Precise, (low). R Between 2 and 3 samples.	Scaffold MSQuant Elucidator ASAPRatio	http://www.proteomesoftware.com/ http://msquant.sourceforge.net/ http://www.rosettabio.com/ http://tools.proteomecenter.org/
Isotopic labeling					
ICAT, ^18^O, ICPL	1 to 2	Poor	Precise, (low). R Between 2 conditions.	Elucidator XPRESS [27] MSQuant ASAPRatio [28] ZoomQuant [29]	http://www.rosettabio.com/ http://tools.proteomecenter.org/ http://msquant.sourceforge.net/ http://tools.proteomecenter.org/ http://proteomics.mcw.edu/zoomquant.html
Isobaric labeling					
ITRAQ, TMT, DIGE	2 3	Medium	Medium, (low). R or A Between 2 and 8 conditions.	ProteinPilot Multi-Q [30] iTracker [31] MSQuant	http://www.absciex.com/ http://ms.iis.sinica.edu.tw/Multi-Q-Web/ http://www.cranfield.ac.uk/ http://msquant.sourceforge.net/
Targeted					
MRM Isotope dilution +/− heavy label	5 Attomolar detection.	Poor^1^	Precise, (high). R or A Requires intensive method development.	Skyline [32] MaxQuant ATAQS [33] MRMer [34]	https://brendanx-uw1.gs.washington.edu http://maxquant.org/ http://tools.proteomecenter.org/ATAQS/ATAQS.html http://proteomics.fhcrc.org/CPL/MRMer.html

^a^Orders of magnitude.

APEX: absolute protein expression profiling. emPAI: exponentially modified protein abundance index. SILAC: stable isotope labelling by amino acids. DIGE: Difference Gel Electrophoresis. ICAT: isotope-coded affinity tags. ITRAQ: isobaric tags for absolute and relative quantitation. TMT: tandem mass tags. MRM: multiple reaction monitoring.

^1^Few target proteins can be selected efficiently in a single LC-MS/MS experiment.

MS^2^: MSMS

**Table 2:** Recent quantitative MS-based studies involving human samples.

Authors/year	Specimen	Quantitative approach	Sample preparation	Outcomes
Yang et al. 2011 [46]	Urine 54 bladder cancer patients, 46 controls	Label-free—spectral count	NIL	Quantified 265 glycoproteins. alpha-1-antitrypsin, 74% sensitivity and 80% specificity for bladder cancer patients.
Quintana et al. 2009 [47]	Urine 39 patients kidney chronic allograft dysfunction, 32 controls	Label-free—peak peptide intensity	SCX using magnetic beads	Peptides from uromodulin and kininogen significantly elevated in control compared to CAD patients.
Hanas et al. 2008 [48]	Serum 13 pancreatic adenocarcinoma patients, 12 healthy controls	Label-free—peak peptide intensity	NIL	Quantified 20 low-mass serum peaks. Bootstrap analysis showed peaks could differentiate cancer from control sera with 95% accuracy.
Xue et al. 2010 [49]	Cell lysates Primary and lymph node metastatic cell lines. 1 patient.	Label-free—peak peptide intensity	NIL	145 differential proteins. Western blot and ROC curve analysis confirmed that 2 specific proteins could predict colorectal cancer metastasis.
Besson et al. 2011 [50]	Colorectal cancer tissue 28 colorectal frozen tissue samples	Stable isotope labeling—iTRAQ	Peptide OFFGEL fractionation	555 proteins with significant fold change between different cancer stages. Identified a candidate with increased abundance in adenomas and early stage colorectal cancer.
Bondar et al. 2007 [51]	Serum 6 healthy male, 20 nonmalignant prostate biopsy patients, 26 malignant prostate cancer patients	Stable isotope labeling	NIL	Higher abundance of Zn-*α*2 glycoprotein (ZAG) in prostate cancer patients than nonmalignant prostate disease patients and healthy controls.
Chaerkady et al. 2008 [52]	Liver tissue 55 samples of hepatocellular carcinoma, 20 samples of adjacent noncancer tissues	Stable isotope labeling—iTRAQ	SCX	59 proteins increased in abundant, 92 proteins were less abundant in HCC compared to normal tissue. 12 proteins further validated using immunohistochemical labeling.
Dayon et al. 2008 [53]	Cerebrospinal fluid 4 postmortem CSF patients, 4 antemortem CSF from living healthy controls	Stable isotope labeling—tandem mass tag isobaric labeling	Immunoaffinity depletion of 6 most abundant proteins and SCX	78 proteins more abundant in postmortem samples compared to antemortem.

**Table 3:** Summary of MRM quantitative analysis in blood for a variety disease types.

Authors/year	Specimen	Target	Sample preparation	MS platform	Outcomes
Stahl-Zeng et al. 2007 [65]	Plasma	N-glycoproteins	Selective isolation of N-glycosites. Stable isotope ^13^C- and/or ^15^N-labelled reference peptides.	LC ESI MS/MS Hybrid triple quadrupole linear ion trap	Detection ≤ ng/mL concentration range and accurate quantification over a linear range of ~ 10^5^. LOQ of 50 amol. LOD ≥ 10 amol. Protein concentration in plasma of 0.1 ng/mL.
Anderson and Hunter 2006 [66]	Plasma 1 healthy donor	53 plasma proteins	Top six abundant proteins depleted. Stable isotope labeled internal standards.	ESI LC-MS/MS 4000 Q Trap Hybrid triple quadrupole/linear ion trap	Quantitative data for 47 proteins in the *µ*g/mL level over linear range of 10^4^
Keshishian et al. 2007 [67]	Plasma 1 healthy donor	6 low abundance plasma proteins	Abundant protein depletion and SCX chromatography. Stable isotope-labeled amino acids.	ESI LC-MS/MS 4000 Q Trap Hybrid triple quadrupole/linear ion trap instrument	LOQ of 1–10 ng/mL range and linearity ≥ 10^2^. LOD in high pg/mL.
McKay et al. 2007 [68]	Plasma 4 colorectal cancer patients undergoing chemotherapy	18 liver-derived proteins in plasma	Immunodepletion (Albumin and IgG removed)	ESI LC-MS/MS 4000 Q Trap Hybrid triple quadrupole/linear ion trap instrument	Increase in target plasma proteins during treatment. Similar trends found in MRM assays and 2-D DIGE
Kirsch et al. 2007 [69]	Blood bank pooled serum	2 human growth hormones (IGFBP-3, IGF-1)	NIL	ESI LC-MS/MS Triple quadrupole mass spectrometer	Detection ranges of 4–10 ng/*µ*l for IGFBP-3 and 2–8 ng/*µ*l for IGF-1.
Kuhn et al. 2004 [70]	Serum. Pools of healthy, non-erosive RA and erosive RA ( 5 individuals per pool).	C-reactive protein	Immunodepletion of haptoglobin, IgG and HSA, then size exclusion chromatography	ESI LC-MS/MS Triple quadrupole mass spectrometer	Correlation between erosive RA, RA and increased CRP over healthy patients. Results verified using immunoassay.
Fortin et al. 2009 [71]	Serum Benign prostate hyperplasia and prostate cancer.	PSA	Immunodepletion of albumin and mixed cation exchange peptide fractionation	ESI LC-MS/MS API 2000 triple quadrupole or 4000 Q Trap hybrid triple quadrupole/linear ion trap	Absolute quant. of PSA to low ng/mL, with good correlation to clinical ELISA tests.
Huillet et al. 2012 [72]	Serum Clinical samples from 5 myocardial infarction patients	Clinically validated cardiovascular biomarkers (LDH-B, CKMB, myoglobin, troponin I)	Immunodepletion of six highest abundant proteins and SDS-PAGE Immunocapture prefractionation and SDS-PAGE	ESI LC-MS/MS 5500 Q Trap hybrid triple quadrupole/linear ion trap mass spectrometer	Absolute quant. using Protein Standard Absolute Quantification (PSAQ) and MRM. Demonstrated good correlation with ELISA assay results.
Zhao et al. 2010 [73]	Serum 10 hepatocellular carcinoma patients and 10 healthy donors	Candidate biomarkers of hepatocellular carcinoma (vitronectin and clusterin)	NIL	ESI LC-MS/MS 4000 Q Trap Hybrid triple quadrupole/linear ion trap instrument	Stable isotope dilution-MRM using 18O-labelling method demonstrated significant downregulated in HCC compared to healthy group. Results comparable to ELISA.
Kuhn et al. 2009 [74]	Plasma 5 patients undergoing PMI and alcohol ablation treatment for HOCM	Troponin I, and Interleukin 33	Immunoaffinity enrichment SISCAPA	ESI LC-MS/MS Triple quadrupole instrument	Linearity from1.5 to 5000 *µ*g/L and correlated with commercial immunoassay. Demonstrated how SISCAPA-MRM can quantify changes to low *µ*g/L levels.
Lopez et al. 2011 [75]	Serum from 24 trisomy 21 and 21 normal first trimester pregnancies	12 putative markers of Trisomy 21	NIL	ESI LC-MS/MS LTQ Orbitrap XL mass spectrometer	Developed a workflow for Trisomy 21. Protein biomarkers targeted are high abundance proteins. SRM LOQ of 1–5 femtomoles..
Domanski et al. 2012 [76]	Plasma 90 patients with cardiovascular disease	67 putative markers of cardiovascular disease	NIL	ESI LC-MS/MS Agilent 6490 triple Quadrupole LC/MS	117 from 135 peptides with attomolar LOQ for 81 peptides.
